# Simulation Analysis of Cluster Effect of High-Shear Low-Pressure Grinding with Flexible Abrasive Tools

**DOI:** 10.3390/mi12070827

**Published:** 2021-07-15

**Authors:** Chengjin Tian, Jinguo Han, Yebing Tian, Bing Liu, Zhiqiang Gu, Xintao Hu

**Affiliations:** 1 School of Mechanical Engineering, Shandong University of Technology, Zibo 255049, China; zbtiancj@163.com (C.T.); hankeyee@163.com (J.H.); liub384444503@163.com (B.L.); dezay520@163.com (Z.G.); 17853311158@163.com (X.H.); 2 Institute for Advanced Manufacturing, Shandong University of Technology, Zibo 255049, China

**Keywords:** flexible grinding, shear thickening fluid, cluster effect, high-shear low-pressure

## Abstract

Based on the clustering effect of shear-thickening fluids (STFs), a high-shear low-pressure flexible grinding wheel has been developed. In order to explore the material removal mechanism, the coupled Eulerian—Lagrangian (CEL) method is adopted to simulate the novel grinding process. The simulation results show that particle clustering effects do occur at the tangential and bottom positions of the micro-convex peak when it instantaneously strikes the workpiece surface. The particle clusters drive the harder abrasive particles to resist the strong interactions of micro-convex peaks. The micro-convex peaks are removed due to the cutting effect of the harder abrasive particles. Compared with traditional grinding, the ratio of tangential force to normal force for the high-shear low-pressure flexible grinding wheel is improved. The various trends in force ratio are consistent with the experimental results, which verifies the effectiveness of high-shear low-pressure grinding.

## 1. Introduction

With the continuous development of science and technology, an increasing number of difficult-to-process materials with excellent performance have appeared in the aerospace industry, medical equipment manufacturing, automobile manufacturing, precision manufacturing and other industries [1,2,3,4]. Due to the improvement in requirements for material quality and surface accuracy within these industries, some difficult-to-process materials require further processing before they can be used. Grinding is the most widely used precision and ultra-precision processing technology, and is commonly employed in the aerospace industry, precision manufacturing and other fields [5,6,7,8]. Grinding is a multi-edge composite cutting technology. The large negative rake angle and the arc radius of the cutting edge drive elastic deformation of the surface of the workpiece [9]. As the cutting depth continues to increase, the surface of the workpiece will form abrasive debris along the cutting direction, which will fall away from the surface of the workpiece. A grain interacts with the workpiece in three stages of material deformation, i.e., sliding, ploughing, and cutting [10]. It is well known that the grinding force plays an important role in the grinding process, and can be divided into the tangential grinding force, normal grinding force and axial grinding force. Generally, the normal grinding force is 2–3 times larger than the tangential grinding force in the traditional grinding process [11]. For aerospace parts and hard-brittle composite materials, this ratio can increase to hundreds of times, which makes the process prone to a series of grinding problems such as grinding cracks, grinding burns, surface and subsurface defects, etc. [12].

In response to the aforementioned problems, many scholars have developed various new grinding wheels, such as the ordered abrasive grain grinding wheel [13], the grooved grinding wheel [14] and the single-layer brazing grinding wheel [15]. Both the grooved grinding wheel and the ordered grinding wheel can improve processing quality and reduce the normal grinding force. However, the grooved grinding wheel will affect the smoothness of its processing due to its non-continuous surface characteristics. Additionally, the ordered abrasive grain grinding wheel is still in the early stage of research, which means that the abrasive grain arrangement efficiency is relatively low. Compared with traditional grinding wheels, single-layer brazing grinding wheels have many advantages. Nevertheless, the grinding working layer is relatively thin, which means that the service life is relatively short. Zhang et al. [16] developed a diamond grinding wheel with a structured surface. Through the grinding of zirconia ceramic parts, it was found that the new surface-textured diamond grinding wheel forms a coolant film during the grinding movement to improve the quality of the processed table and reduce the grinding force. Wu et al. [17] developed a combined brazed diamond sheets and original resin grinding wheel. The experimental results showed that with increasing working load, the wear rate of the brazing sheet increased and the grinding ratio decreased. In addition, the temperature rose faster. Teicher et al. [18] used a single-layer fiber-welded grinding wheel to perform grinding experiments on titanium alloys. The grinding quality improved when the alkaline grinding fluid was added. Tian et al. [19] used selective laser melting to design metal-bonded diamond grinding wheels, where both improved surface roughness and reduced work hardening were achieved in comparison to electroplated wheels. Tour et al. [20] investigated a grinding wheel made of carbon-fiber-reinforced plastic (CFRP). In comparison with traditional steel wheels with different fiber directions, CFRP wheels have higher anti-vibration stability. Although the grinding wheel made of CFRP can reduce the centrifugal force and thermal stress deformation, the production cost of the CFRP grinding wheel is relatively high. The aforementioned grinding tools are characterized as consolidated abrasive grinding wheels. They have been verified as insufficiently processing some complex profiles. Therefore, great efforts are underway to develop compliant abrasive technologies with enhanced self-adaptability of grinding tools. Islam [21] and Becaucamp [22] developed a bonnet-polishing method based on an elastic tool, which can adapt to the flat surface and freeform better. With further development of compliant abrasive technologies, Walker et al. [23] proposed a shape adaptive grinding (SAG) method using a fabric sheet deposited with diamond grains. The surface roughness was reduced to less than 2 nm. At present, compliant abrasive technologies have become one of the most promising methods for complex profile finishing.

In previous work [5,7], we used STF-Kevlar composite materials with abrasive grains to make a flexible grinding wheel. However, it was difficult to verify the grinding mechanism of the developed high-shear low-pressure flexible grinding wheel in experiments. Thus, the simulation method was adopted to explore the grinding mechanism of the high-shear low-pressure flexible grinding with the flexible STF-Kevlar composited wheel. The processing model of the high-shear low-pressure flexible grinding was simplified as micro-convex peaks impacting the surface of the flexible grinding wheel. Many researchers have already explored the STF-Kevlar composite materials through simulation. Sen et al. [24] used CEL to explore the impact resistance of Kevlar-STF composites. The simulation results showed that the lateral deformation, energy distribution, etc., of the Kevlar-STF composite materials were significantly improved compared with the current multi-layer pure Kevlar. The viscosity of the STF consumed approximately 75% of the energy. Mahdi et al. [25] used LS-DYNA to simulate the impact of Kevlar impregnated with STF. The effect of STF on Kevlar fiber was simulated by changing the friction coefficient of Kevlar warp and weft yarn. Moreover, the friction coefficient of Kevlar fiber and impact body was also changed. The simulation results showed that the Kevlar impregnated with STF improved the friction coefficient between the yarns, thereby improving the impact resistance of the Kevlar fiber. Lu et al. [26] explored the impact properties of warp knitted spacer fabrics (WKSFs) impregnated with STF by simulation. The simulation results showed that the energy absorption of WKSF/STF composite materials occurred in the thickening stage of STF and the friction between yarns. In this paper, the CEL method was adopted to simulate the grinding process and explore the cluster effect of STF. The simulation results showed that, due to the instantaneous impact of the micro-convex peaks on the surface of the workpiece, particle cluster effects are likely to occur at the tangential and bottom positions of the micro-convex peaks. Compared with traditional grinding, the ratio of tangential force to normal force of the high-shear low-pressure grinding is improved.

## 2. Principle of High-Shear and Low-Pressure Grinding

**Figure 1:**
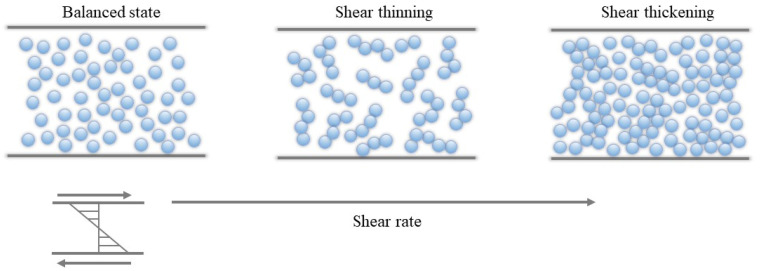
The mechanism of shear-thickening cluster effect.

**Figure 2:**
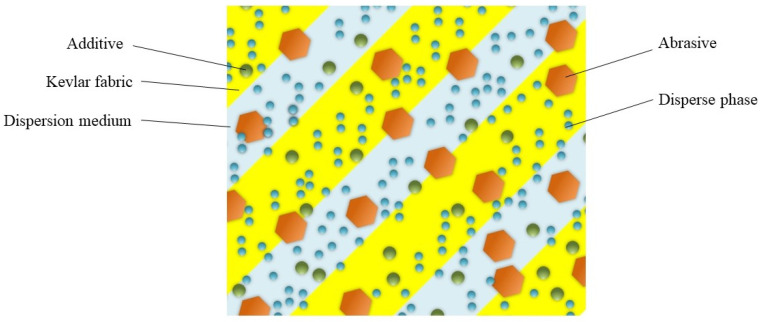
Composition of the high-shear low-pressure grinding wheel.

To address the problem that the ratio of tangential grinding force to normal force is too large, this paper developed a high-shear low-pressure flexible grinding wheel. The high-shear low-pressure flexible grinding wheel was made by adding shear thickening fluid to Kevlar fiber. Some dispersed phase nanoparticles were also mixed. The mechanism of the shear thickening cluster effect is shown in Figure 1. Generally, shear-thickening fluids are liquid when in a balanced state. However, when suffering an instantaneous impact, the shear rate increases rapidly. The dispersed phase particles will undergo localized thinning. As the shear rate increases, dispersed phase nanoparticles will quickly aggregate to produce a cluster effect and further form a particle cluster effect. The instantaneous impact makes the shear thickening flow fluid instantaneously turn into a solid-like state to absorb the impact kinetic energy. A new type of flexible grinding wheel was fabricated in this research by employing the cluster effect of the shear thickening fluid. Figure 2 illustrates the composition of flexible grinding where dispersed medium, dispersed phase, Kevlar fiber, abrasive particles, and additives are included. 

**Figure 3:**
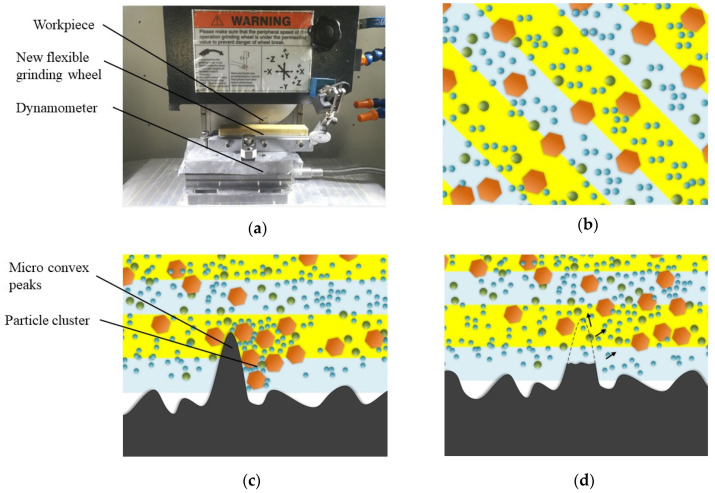
The processing principle of the high-shear low-pressure flexible grinding wheel. (**a**) Experimental setup. (**b**) High-shear low-pressure flexible abrasive grinding wheel in balanced state. (**c**) High-shear low- pressure flexible grinding wheel contact the workpiece and cluster effect occurs. (**d**) The micro-convex peaks are removed and return to the initial state.

The processing principle of the high-shear low-pressure flexible grinding wheel is shown in Figure 3. The experimental setup is introduced by Figure 3a. The high-shear low-pressure flexible grinding wheel is loaded on the fixture. the workpiece is fixed and impacts the high-shear low-pressure flexible grinding wheel. The dynamometer is used to monitor the grinding force and indicate the force ratio. In the initial stage of processing, as shown in Figure 3b, the dispersed phase and abrasive particles are uniformly dispersed in the shear-thickening liquid. In Figure 3c, when the grinding wheel touches the micro-convex peaks, the micro-convex peaks produce an instantaneous impact on the grinding wheel and break the balance state. Subsequently, the shear rate increases and the dispersed phase nanoparticles quickly aggregate to form a cluster. The viscosity of the fluid continues to increase, showing a solid-like state. At the same time, the abrasive particles are quickly gathered by the dispersed phase nanoparticles, so that a large number of abrasive particles collide with the micro-convex peaks to achieve the purpose of material removal. In the recovery stage of Figure 3d, the micro-convex peaks are removed. The impact load on the surface of the flexible abrasive grinding wheel disappears. At this time, the dispersed phase nanoparticles are rapidly dispersed into the dispersion medium. The cluster effect is continuously weakened and the viscosity continues to decrease. The abrasive particles are also uniformly dispersed in the dispersion medium along with the dispersed phase nanoparticles. As the grinding process continues, this process proceeds cyclically to achieve material removal. 

## 3. Numerical Simulation Model

### 3.1. STF Model Establishment and Material Parameter Definition

The processing model of the high-shear low-pressure flexible grinding is simplified, as micro-convex peaks impact the surface of the flexible grinding wheel. Due to the short impact time of the model, it can be regarded as a transient explicit dynamic simulation and must be adapted to the large deformation of the STF. The CEL technology combining Lagrangian and Eulerian grids is mostly used for fluid–structure coupling analysis. The model of STF grinding is regarded as a fluid–solid coupling model. STF usually exhibits large motion deformation under low viscosity. Therefore, CEL technology is used to simulate the high-shear low-pressure flexible grinding wheel. The Mie-Grüneisen equation of state (EOS) is used to define its viscosity state. Abrasive particles are added to the STF to study the cluster effect of STF and the influence of grinding force. The Mie–Grüneisen equation of state (EOS) describing the behavior of STF fluid is shown below:

(1) $$p=\frac{\rho_{0}C_{0}^{2}x}{{\left(1-sx\right)}^{2}}\left(1-\frac{\Gamma_{0}x}{2}\right)+\Gamma_{0}E$$

where p is the pressure, $C_{0}$ is the speed of sound through the medium, $\rho_{0}$ is the initial density, ρ is the current density, $x=\left(1-\frac{\rho_{0}}{\rho }\right)$, $s=dU_{s\;}/dU_{p}$ is a linear Hugoniot slope coefficient, $\Gamma_{0}$ is material constant, $U_{s}$ is the shock wave transmission speed and $U_{p}$ is the particle speed. In ABAQUS, the fluid behavior of STF can be defined by the $U_{s}$-$U_{P}$ state equation. Table 1 shows the material parameters of STF [24].

**Figure 4:**
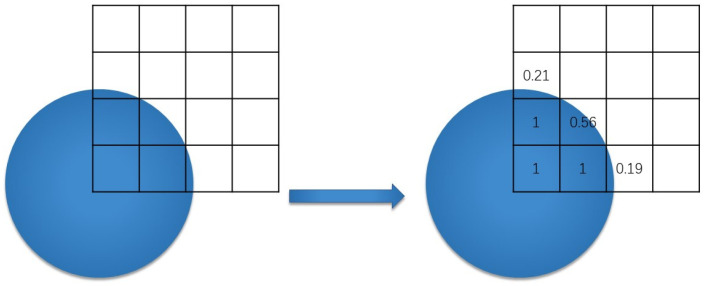
Principle of Eulerian Analysis.

Due to the fluid characteristics of STF itself, it cannot be analyzed in a traditional way. This simulation adopts the method of CEL analysis. Eulerian analysis is used to fix the Eulerian grid so the material can flow freely within it. In each time increment, the proportion of the grid occupied by the material is calculated as shown in Figure 4. If the material completely occupies the grid, EVF = 1, and if the material does not occupy the grid, it is 0.

**Figure 5:**
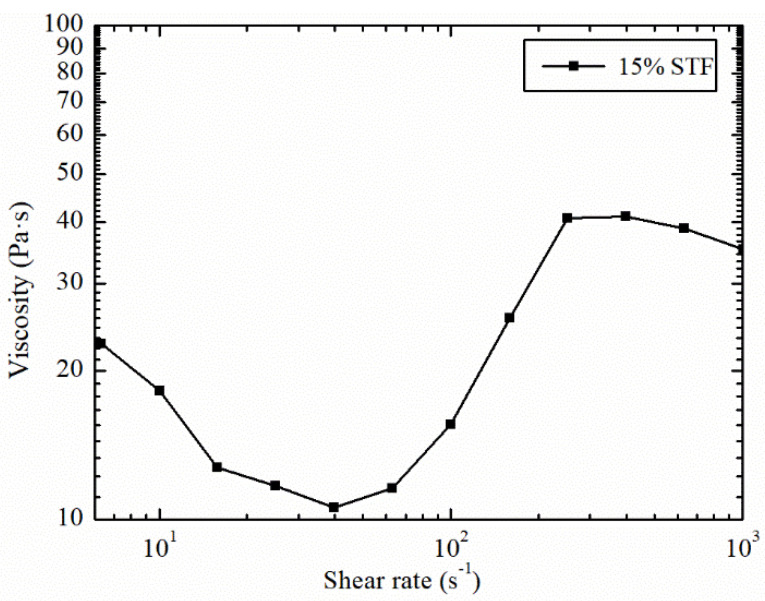
Rheological performance of 15% mass fraction STF.

In our experiments, a non-Newtonian fluid was prepared by PEG200 and SiO_2_. PEG200 and 15% mass fraction of SiO_2_ were mixed in a 60 °C oil bath and abrasive grains were added. The rheological performance of PEG200/SiO_2_ non-Newtonian fluid was tested and the result was shown in Figure 5.

It can be seen from Figure 5 that as the shear rate continues to increase, the shear-thickening effect is gradually strengthened, reaching a peak viscosity of 41.1 Pa·s. According to the literature [24], the impact velocity can be divided by the diameter of the micro-convex peak. Therefore, the shear rate of the micro-convex peaks to the composite model can be roughly measured. It is estimated that the lowest shear rate (5000 s^−1^) is much higher than the shear rate (400 s^−1^) of the peak viscosity in Figure 5, so the viscosity of STF is set at 40 Pa·s. At the same time, different viscosity gradients (10 Pa·s, 20 Pa·s, 30 Pa·s) are used for comparison. These assumed constant viscosity values at higher strain rates are considered independent of impact velocity.

### 3.2. High-Shear Low-Pressure Flexible Grinding Wheel Composite Model

**Figure 6:**
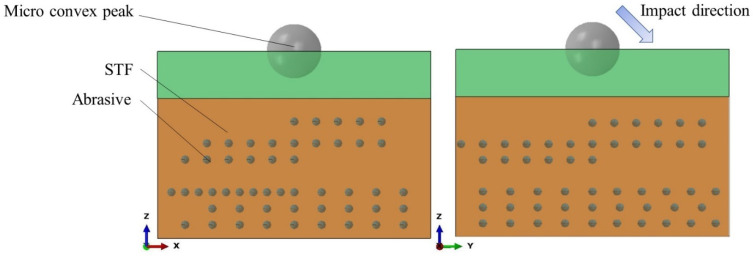
High-shear low-pressure flexible grinding wheel composite model.

In finite-element simulation, a good model can not only ensure the accuracy of the simulation, but also save time. Different from traditional impact simulation [24,25,26], small balls of different sizes are used to simulate the micro-convex peaks and abrasive particles. Compared with the abrasive grains, the hardness of the micro-convex peaks is relatively soft. The abrasive particles and the micro-convex peaks continuously interact to achieve the purpose of material removal. The micro-convex peaks and abrasive grains are modeled by Lagrangian. The element types of the micro-convex peaks and abrasive grains are both C3D8R. In order to accurately and quickly explore the clusters effect on the high-shear low-pressure flexible grinding wheel, the micro-convex peak is set as a rigid body. In addition, the material of abrasive and micro-convex peak are white corundum and steel. The STF is simplified to a rectangular model by Eulerian, and the element type is EC3D8R. At the same time, the overall model is enlarged in order to avoid the problem of the simulation not being easy to converge due to its small scale. Figure 6 is the high-shear low-pressure flexible grinding wheel composite model. The outermost layer is the established Eulerian body. The established Eulerian body is defined in two layers, and the uppermost layer does not specify any material (equivalent to an air layer). The established micro-convex peak model is embedded into it, the lower layer is defined as STF, and abrasive particles are added to the STF. In the actual processing process, abrasive particles are randomly added to the STF, so the established model also adopts a random distribution of abrasive particles. The impact angle and speed are 45 degrees and 100 m/s, respectively.

## 4. Results and Discussion

### 4.1. Simulation Analysis of Cluster Effect

**Figure 7:**
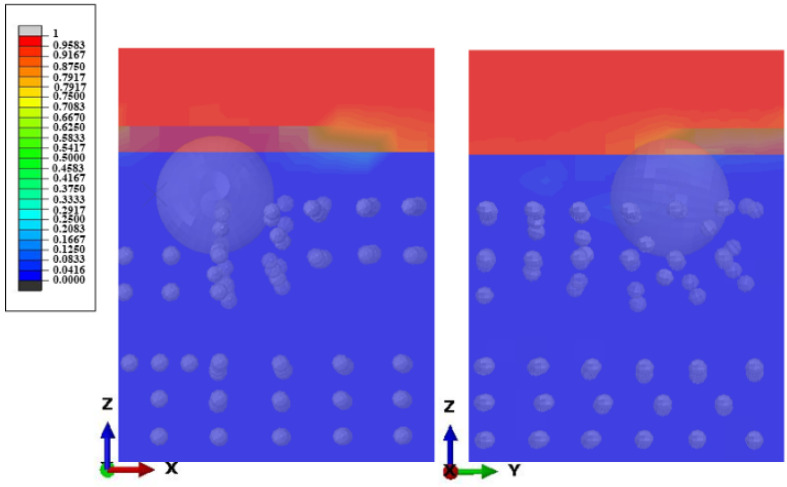
The sectional view of particle clustering under 40 Pa·s viscosity.

Figure 7 shows a cross-sectional view of particle clustering under 40 Pa·s viscosity. It can be seen that when the micro-convex peak made contact with the STF, the surface of the STF fluctuated due to the impact. However, due to its high viscosity, the splashed STF jet did not disperse. As the impact of the micro-convex peak continued to deepen, an air cavity was formed in the path of the impact of the micro-convex peaks. The abrasive particles began to make contact with the micro-convex peak, and gradually created a particle clustering effect. The particle clusters drove the abrasive particles to accumulate in the tangential direction and bottom of the micro-convex peak, so that the micro-convex peak and the aggregated abrasive particles were continuously in contact. Eventually, this achieved the purpose of removing the micro-convex peak.

**Figure 8:**
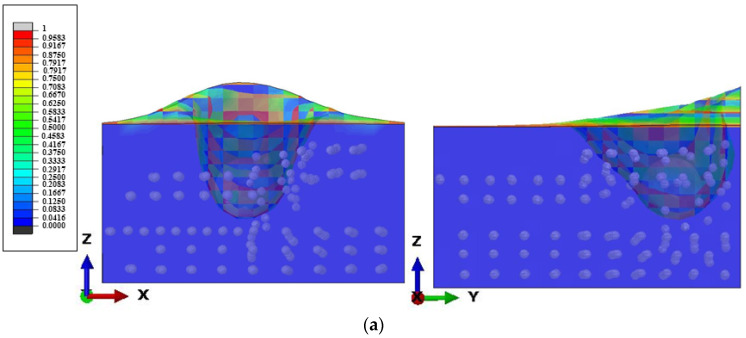

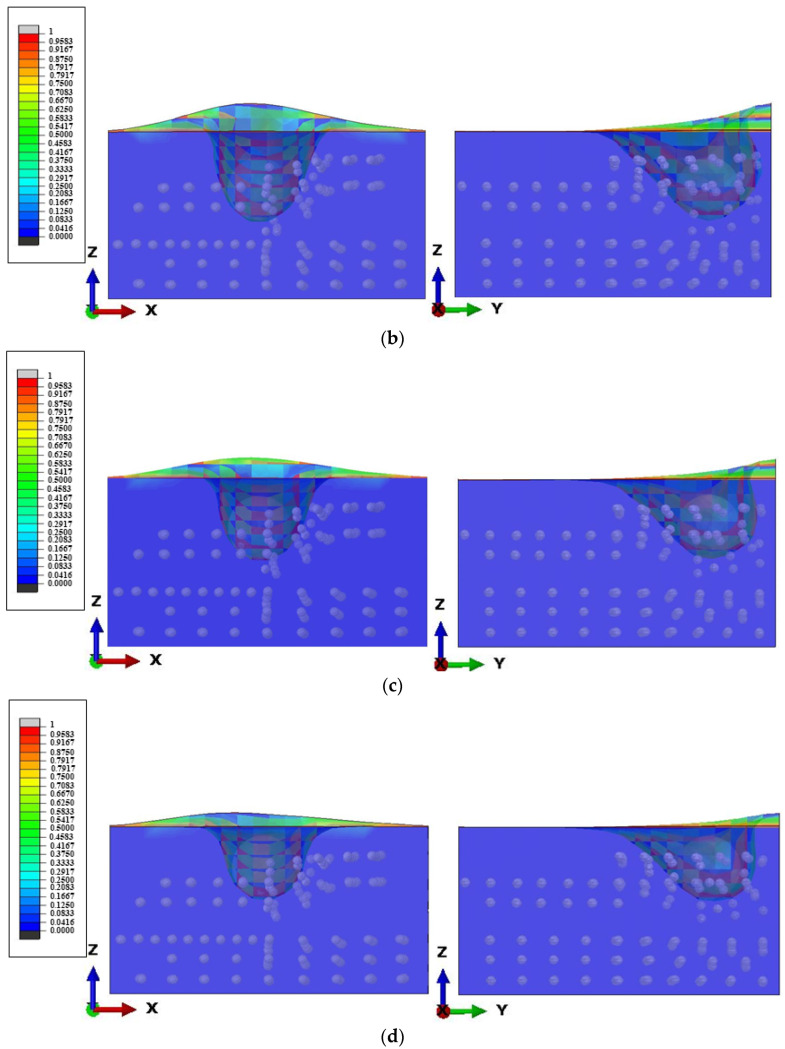
Particle clustering effect under (**a**) 10 Pa·s viscosity, (**b**) 20 Pa·s viscosity, (**c**) 30 Pa·s viscosity, (**d**) 40 Pa·s viscosity.

Figure 8 shows the results of particle clusters at 40 Pa·s, 30 Pa·s, 20 Pa·s, 10 Pa·s viscosities. It can be seen from the figure that the surface of the STF also fluctuated due to the impact after the micro-convex peak made contact with the STF. However, the fluctuation height was different under different viscosities. The highest jet of STF occurred at a viscosity of 10 Pa·s, and the lowest at 40 Pa·s. The particles under different viscosities had a clustering effect, which made the abrasive particles agglomerate. However, the particle clustering at the impact of 40 Pa·s viscosity was more concentrated. This shows that the higher the viscosity, the more obvious the clustering effect of particles, which can achieve a good material removal effect. In the existing literature, the theories explaining the shear thickening behavior of STF mainly include order–disorder theory [27] and particle cluster theory [28]. Both theories discuss the shear-thickening behavior from the microscopic particle scale. According to the theory of fluid mechanics, the shear-thickening behavior is due to the mutual dynamic lubrication between particles [29]. When the STF is instantaneously impacted, the shear force in the STF increases instantaneously, causing the dispersed particles to collide with each other. The dynamic pressure in the fluid increases instantaneously, which manifests as an instantaneous increase in the macroscopic viscosity of the STF.

### 4.2. Analysis of Tangential Grinding Force and Normal Grinding Force

**Figure 9:**
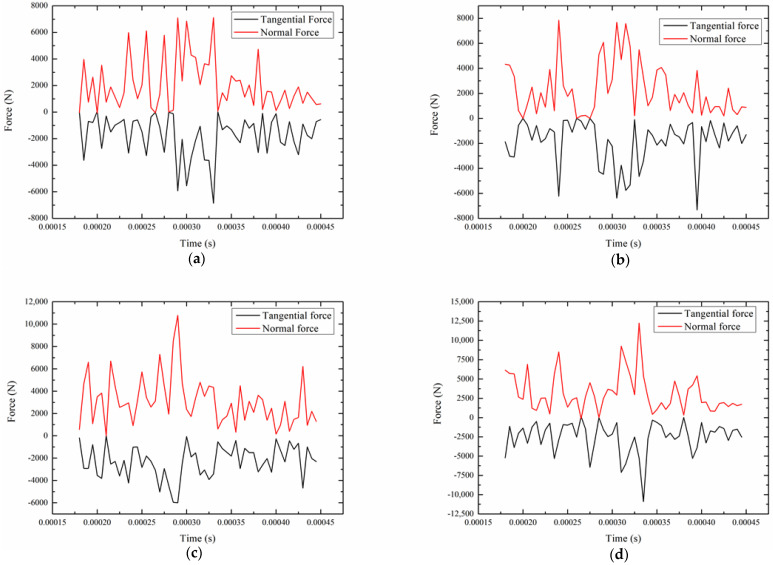
Normal force and tangential force values under different viscosities (**a**) 10 Pa·s viscosity, (**b**) 20 Pa·s viscosity, (**c**) 30 Pa·s viscosity, (**d**) 40 Pa·s viscosity.

In the grinding process, the grinding force has a vital influence on the grinding quality and grinding efficiency. In traditional grinding, the normal force is usually 2–3 times larger than the tangential force [11,30,31]. The clustering effect of STF can cause abrasive particles to gather at the tangential position under the tangential impact. Compared with the normal force, the tangential force has a more obvious increase, which improves the force ratio of tangential force to normal force to achieve the effect of high-shear low-pressure. Figure 9 is the numerical values of normal force and tangential force under different viscosities. It can be seen from Figure 9 that the tangential force and the normal force reach their peak values at 0.0003–0.00035 s. Moreover, the peak values of the tangential force and the normal force are not very different under each viscosity. As time goes by, the tangential force and the normal force gradually decrease. However, a convex peak appeared at a viscosity of 20 Pa·s, which is caused by the extrusion and collision of some abrasive grains on the micro-convex peak. The average value of the tangential force and the normal force under each viscosity was stable at 2000–3000 N. In addition, the tangential force and the normal force show a periodic upward and downward trend. In the early stage of impact, the peak values of the tangential force and the normal force at each viscosity are relatively small and stable at 3800 N. As the impact continues to deepen, abrasive particles increasingly gather at the tangential and bottom positions of the micro-convex peak. The abrasive particles continuously collide with the micro-convex peak, resulting in an increase in the peak values of tangential force and normal force. At the end of the impact, due to the continuous action of the clustering effect, the kinetic energy of the micro-convex peaks is continuously reduced. Finally, the degree of collision and extrusion with the abrasive particles is reduced.

**Figure 10:**
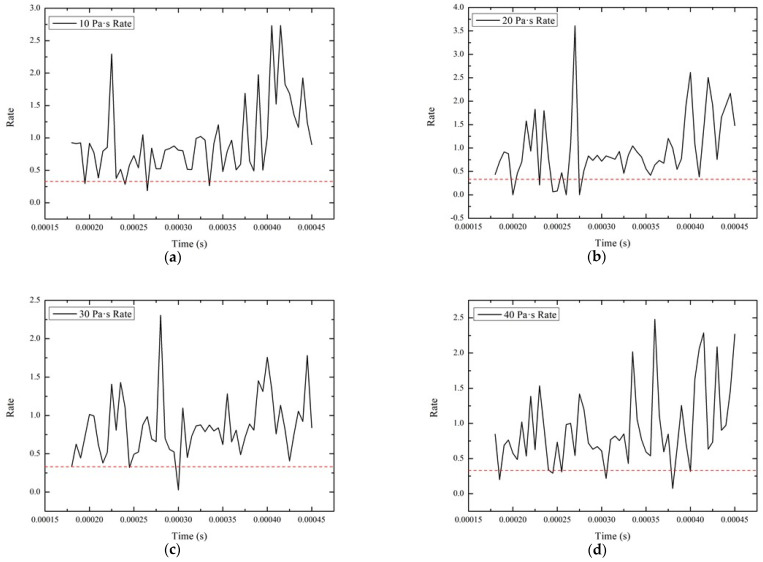
Force ratio of the tangential force to the normal force under different viscosities (**a**) 10 Pa·s viscosity, (**b**) 20 Pa·s viscosity, (**c**) 30 Pa·s viscosity, (**d**) 40 Pa·s viscosity.

In order to analyze the effect of the high-shear low-pressure flexible grinding wheel more intuitively and accurately, the ratio of the tangential force to the normal force at each time point is plotted, as shown in Figure 10. The ratio of the tangential force to the normal force at each viscosity is mostly greater than 0.33 (the position of the red dotted line in the Figure 10). The peak force ratio of some viscosities reach 2.5–3.2. Moreover, the average value of the force ratio reaches the maximum value of 0.82 at 40 Pa·s. The simulation results were compared with the experimental results of Tian and Li [32]. Under the experimental conditions, the ratio of the tangential force to the normal force under the pure Kevlar fiber grinding condition is 0.357, and the ratio of the tangential force to the normal force is increased to 1.055 by adopting the high-shear low-pressure flexible grinding wheel. The tangential force and the normal force ratio trend are consistent with the experimental results, which verifies the effectiveness of high-shear low-pressure grinding. The results show that STF can produce a clustering effect when subjected to a high-speed impact, which make the dispersed phase particles agglomerate in large numbers from a micro perspective. From a macro perspective, the high-shear low-pressure flexible grinding wheel can effectively increase the force ratio between the tangential force and the normal force.

### 4.3. Speed Analysis

**Figure 11:**
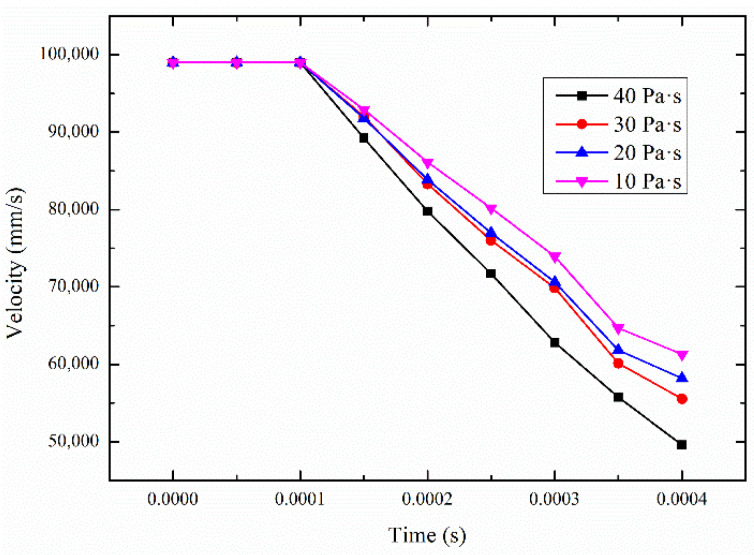
Speed changes rate in different viscosities.

The clustering effect of STF can not only bring about the high-shear low-pressure grinding effect, but can also absorb the impact kinetic energy of the micro-peaks and reduce the impact speed of the micro-convex peak. The speed changes at different viscosities are shown in Figure 11. Due to the clustering effect of STF, the speed of the micro-convex peak decreases significantly, which indicates that the kinetic energy absorption effect of STF is more significant. The speed changes rate between the viscosities of 10 Pa·s and 20 Pa·s is large. However, the rate between the viscosities of 20 Pa·s and 30 Pa·s is relatively small. As the viscosity increases, the rate of speed change gradually increases. The biggest rate is between the viscosities of 30 Pa·s and 40 Pa·s. When the viscosity is 40 Pa·s, the rate of speed change reaches the maximum. As the impact energy of the micro-convex peak on the STF gradually fades, the cluster effect gradually disappears, and the aggregation effect of the abrasive particles also slowly dissipates. Therefore, the decrease in the speed of the micro-convex peak starts to slow down after 0.00035 s.

## 5. Conclusions

In order to explore the grinding mechanism of the high-shear low-pressure flexible grinding wheel developed here, the processing model of the high-shear low-pressure flexible grinding wheel was simplified as micro-convex peaks impacting the surface of the flexible grinding wheel. The CEL method was adopted for modeling and analysis. The STF was modeled by Eulerian, and the abrasive particles was modeled by Lagrangian. The abrasive grains were embedded in the STF.

Simulation results showed that when the STF is impacted by the micro-convex peak, a cluster effect occurs in the tangential direction and at the bottom. Abrasive particles then quickly gather. The ratio of the tangential force to the normal force during the grinding process was approximately 0.82, which is much higher than the ratio in the traditional grinding process. These results show that the flexible grinding wheel has a good high-shear low-pressure grinding effect.

## Figures and Tables

**Table 1:** Material parameters for STF.

Density (kg/m^3^)	$C_{0}(m/s)$	*s*	$\Gamma_{0}$
2722	2100	3.75	0.8
